# Counteracting bone fragility with human amniotic mesenchymal stem cells

**DOI:** 10.1038/srep39656

**Published:** 2016-12-20

**Authors:** Anna M. Ranzoni, Michelangelo Corcelli, Kwan-Leong Hau, Jemma G. Kerns, Maximilien Vanleene, Sandra Shefelbine, Gemma N. Jones, Dafni Moschidou, Benan Dala-Ali, Allen E. Goodship, Paolo De Coppi, Timothy R. Arnett, Pascale V. Guillot

**Affiliations:** 1 Institute for Women’s Health, University College London, London, UK; 2 Lancaster Medical School, Lancaster University, Lancaster, UK; 3 ONCOLille, Regional University Hospital of Lille, Lille, France; 4 Department of Mechanical and Industrial Engineering, Northeastern University, Boston MA, USA; 5 Institute of Reproduction and Developmental Biology, Imperial College London, London, UK; 6 Institute of Orthopaedics and Musculoskeletal Science, Royal National Orthopaedic Hospital, University College London, Stanmore, UK; 7 UCL Great Ormond Street Institute of Child Health, University College London, London, UK; 8 Department of Cell & Developmental Biology, University College London, London, UK

## Abstract

The impaired maturation of bone-forming osteoblasts results in reduced bone formation and subsequent bone weakening, which leads to a number of conditions such as osteogenesis imperfecta (OI). Transplantation of human fetal mesenchymal stem cells has been proposed as skeletal anabolic therapy to enhance bone formation, but the mechanisms underlying the contribution of the donor cells to bone health are poorly understood and require further elucidation. Here, we show that intraperitoneal injection of human amniotic mesenchymal stem cells (AFSCs) into a mouse model of OI (*oim* mice) reduced fracture susceptibility, increased bone strength, improved bone quality and micro-architecture, normalised bone remodelling and reduced TNFα and TGFβ sigalling. Donor cells engrafted into bones and differentiated into osteoblasts but importantly, also promoted endogenous osteogenesis and the maturation of resident osteoblasts. Together, these findings identify AFSC transplantation as a countermeasure to bone fragility. These data have wider implications for bone health and fracture reduction.

Mesenchymal stem/stromal cells (MSCs) are multipotent non-hematopoietic cells initially isolated from the bone marrow and precursors to bone forming osteoblasts^1^. In addition to their osteoblastic potential, MSCs harbour immunosuppressive, anti-apoptotic, anti-fibrotic and anti-inflammatory properties, making them ideal candidates for clinical applications^2^,^3^. MSCs can be found in a variety of tissues throughout development, with fetal MSCs presenting advantageous characteristics compared to their adult counterparts, including higher and broader differentiation potential and smaller size^4^,^5^. The human amniotic fluid contains self-renewing multipotent amniotic MSCs (AFSCs)^6^, which are characterized by their spindle-shape fibroblastic morphology, plastic adherence, expression of the cell surface markers CD105, CD73, CD90, CD19, and absence of expression of CD34, CD45, and CD29^7^,^8^. AFSCs are attractive candidates for cell therapy because they are easily accessible during pregnancy from the surplus of amniocentesis samples and can be used without ethical restriction^9^,^10^,^11^,^12^. They also have a high expansion potential, are non-tumorigenic, tolerogenic, anti-inflammatory and are small enough to pass through capillary beds to reach distant sites of action^13^,^14^,^15^,^16^. Their immunological properties make it possible to use them as universal allogeneic donor^13^,^14^,^15^,^16^. Compared to their adult counterparts, fetal MSCs have longer telomeres, have accumulated fewer genetic mutations and are easier to reprogram to pluripotency^4^.

Human AFSCs have recently emerged as an effective cell source for functional repair of bone defects and bone tissue engineering, producing robust mineralized bone matrix *in vitro* and *in vivo*^17^,^18^,^19^. Here, we compared the mechanical and structural properties of the bones of osteogenesis imperfecta murine (*oim*) mice, some injected with human AFSCs at birth and others with none, and analysed the mechanistic properties of the donor cells. *Oim* mice are characterized by a brittle skeleton as a result of a single point mutation in the collagen type one alpha 2 chain *(Col1α2)* gene, which prevents the production of the protein^20^,^21^,^22^. As a result, the normal heterotrimeric α1[I]_2_α2[I]_1_ collagen molecule is replaced by the homotrimeric α1[I]_3_ one.

Transplantation of fetal and adult MSCs in mouse models of osteogenesis imperfecta (OI) led to a decrease in long bone fracture rate, but failed to improve bone strength^23^,^24^,^25^,^26^. In this work we demonstrate for the first time the capacity of human AFSCs to protect fragile bones by increasing their strength, plasticity and structural properties, and tissue quality. Although a number of observations support the hypothesis that donor cells mediated bone regeneration by direct cell replacement, we found that AFSCs transplantation promoted resident osteoblast maturation, stimulating endogenous osteogenesis and collagen production, thereby restoring the balance of bone remodelling. These results identify AFSCs as an ethical and available source of fetal stem cells that could be used as countermeasure to bone fragility.

## Results

### AFSCs engrafted into bones and expressed osteoblast markers

**Figure 1:**
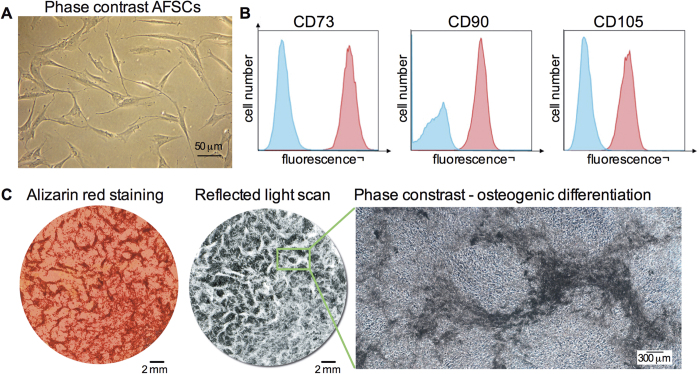
Characterisation of AFSCs. (**A**) Human AFSC morphology *in vitro.* **(B)** Flow cytometry showing population of AFSCs expressing MSC markers CD73, CD90 and CD105 (red). Unstained negative control in blue. **(C)** *In vitro* differentiation of AFSCs down the osteogenic pathways: reflected light scan, alizarin red staining and phase contrast (unstained).

Human mid-trimester AFSCs expressed the stem cell surface marker CD117, adhere to plastic and present spindle-shape morphology (Fig. 1A). The cells complied to the minimal criteria for defining MSCs^1^, i.e. ≥95% of the cell population expressing CD73 (ecto 5′ nucleotidase), CD90 (Thy-1) and CD105 (endoglin) (Fig. 1B), the capacity to differentiate down the adipogenic, chondrogenic and osteogenic pathways (Fig. 1C, Supplementary Figure 1), and lacking expression (≤2%) of CD45, CD34, CD14, CD19 and HLAII (data not shown). AFSCs were thawed in expansion medium, plated at 10^4^ cells/cm^2^ and let to recover for 48 hours before being intraperitoneally infused (10^6^ cells) into *oim* mouse neonates. Donor cell fate was assessed 8 weeks later. All mice injected with AFSCs survived until 8 weeks of age without detectable pathology.

**Figure 2:**
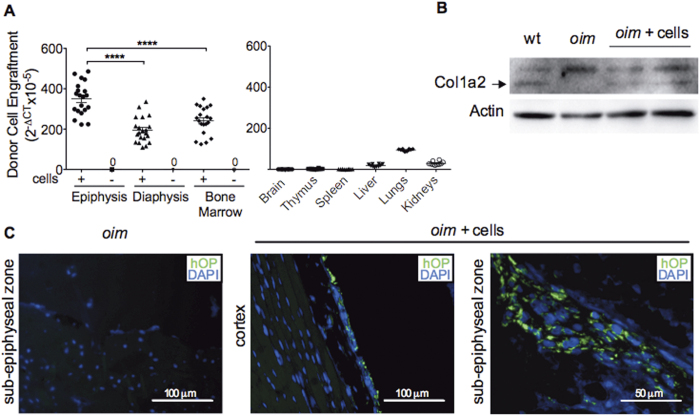
Engraftment of AFSCs. **(A)** Quantitative real time PCR of AFSC engraftment measured as the 2^−ΔCt^ of human specific β-actin normalised to human-mouse non-specific β-actin in the femoral epiphysis (n = 20), diaphysis (n = 20), bone marrow (n = 20), brain (n = 10), thymus (n = 10), spleen (n = 10), liver (n = 10), lungs (n = 10) and kidney (n = 10). P values were calculated using analysis of variance (one-way ANOVA) followed by Bonferroni’s multiple comparison post hoc test. Data represent mean ± SEM. SEM: standard error of the mean. ****P < 0.0001. **(B)** Western blot of collagen type 1 alpha 2 chain (Col1α2) in wild type (wt), non-transplanted *oim* (*oim*) and *oim* mice transplanted with AFSCs (*oim* + cells) (cropped blot is shown, refer to Supplementary information for vision of full blot). **(C)** Immunostaining for human osteopontin (hOP) (green) visualized by fluorescence microscopy in the tibial sub-epiphyseal zone of non-transplanted *oim* (*oim*) and in the tibial sub-epiphyseal zone and cortex of *oim* mice transplanted with AFSCs (*oim* + cells). Nuclei stained with DAPI (blue).

We quantified donor cell engraftment in various tissues using quantitative RT-PCR and primers that amplify human (but not mouse) sequences (hCt) of the housekeeping gene actin, and non-specific primers that amplify both human and mouse sequences (hmCt). Donor AFSCs were detected in bones of all 8 week-old transplanted mice (n = 20). Engraftment levels (2^−DCt^, with ΔCt = hCt-hmCt) in bone epiphysis were 1.8 fold higher than in diaphysis (0.35 × 10^−2^ ± 0.01 vs. 0.19 × 10^−2^ ± 0.01, P < 0.0001) and 1.4 fold higher than in bone marrow (0.24 × 10^−2^ ± 0.01, P < 0.0001). However, the level of donor cell chimerism remained low (average Ct value obtained with human-specific primers ranged from 32 to 34 in bones, and from 18 to 20 for AFSCs in culture). Donor cells were absent in the brain, thymus and spleen, and present at very low levels in liver, lungs and kidneys (Fig. 2A). Osteogenic differentiation of engrafted AFSCs and normalization of the ECM was confirmed by the detection of collagen type 1 alpha 2 chain (Fig. 2B), which is absent in non-transplanted *oim* mice^20^.

We next used a human-specific anti-osteopontin (hOP) monoclonal antibody to determine the differentiation and localization of donor cells *in situ*. Osteopontin is a non-collagenous protein secreted by osteoblasts, which contributes to the formation of the bone extracellular matrix. Staining was localized mainly in the epiphyseal ossification zone, i.e. below the growth plate and, to a lesser extent, in the cortical region (Fig. 2C), indicating that donor cells differentiated down the osteoblast lineage and preferentially homed to sites of active bone formation.

### Intraperitoneal infusion of AFSCs reduced fracture risk

**Figure 3:**
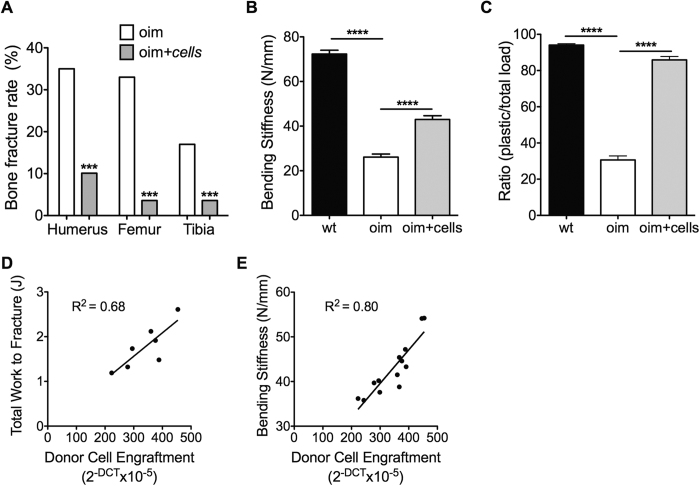
Effect of AFSC transplantation on bone mechanical properties. **(A)** Number of fractured femurs, tibias, and humeri over total number of these bones, assessed by Chi-squared with Yates correction and to one degree of freedom. Differences with a P-value of <0.05 were considered significant. **(B)** Dot-plot of three-point bending load-deflection curves until fracture obtained for 8-week-old wt (n = 20), *oim* (n = 17) and *oim* transplanted (n = 13) femurs for bending stiffness. **(C)** Dot-plot of three-point bending load-deflection curves for ratio of plastic/total load to fracture. Three-point bending data were analysed using analysis of variance (one-way ANOVA) followed by Bonferroni’s multiple comparison post hoc test. **(D)** Linear correlation and regression equation for donor cell engraftment in the femoral epiphysis per mouse against total work to fracture and bending stiffness **(E)**, measured from the three-point bending test. Linear line of best fit is given. Data represent mean ± SEM. SEM: standard error of the mean. ****P < 0.0001, ***P < 0.001, **P < 0.01 and *P < 0.05. wt: wild type non-transplanted mice, oim: non-transplanted *oim* mice, oim + cells: *oim* mice transplanted with AFSCs.

To investigate whether AFSC transplantation reduced long bone fragility, we counted the number of *oim* mice presenting at least one fracture (or fracture callous) in the femur, tibia, and humerus. Whilst 100% of non-transplanted *oim* mice (n = 26) showed at least one long bone fracture, over two-thirds (68%) of transplanted *oim* mice (n = 28) presented no fractures by the age of 8 weeks (Supplementary Figure 2A). The fracture incidence was reduced by 69% in humeri (18/52 vs. 6/56 bones), 89% in femurs (17/52 vs. 2/56 bones); and by 79% in tibias (9/52 vs. 2/56 bones), with an overall of 79% decrease in fracture rate (X^2^ = 28.8, P < 0.0001) (Fig. 3A), indicating that neonatal AFSC infusion prevented fracture occurrence in adulthood.

### AFSCs improved the toughness of the bones

We next used a three-point bending test to determine the mechanical properties of the bones. We found that AFSC transplantation increased the elasticity of *oim* femurs, indicating that they gained deformable properties, i.e. bending stiffness (S) was increased by 64% (72.3 ± 1.7 N/mm vs. 26.1 ± 1.4 N/mm vs. 42.9 ± 1.7 N/mm; mean ± sem, wt vs. *oim* vs. *oim* + cells, P < 0.0001) (Fig. 3B); ultimate load (F_ult_) was increased by 63% (13.7 ± 0.4 N vs. 4.6 ± 0.3 N vs. 7.5 ± 0.4 N, P < 0.0001) (Supplementary Figure 2B); and yield load (F_yield_) was increased by 73% (7.6 ± 0.2 N vs. 3.0 ± 0.2 N vs. 5.2 ± 0.3 N, P < 0.0001) (Supplementary Figure 2C). Compared to the bones of non-transplanted *oim*, the femurs of transplanted *oim* mice were also stronger, as evidenced by an increase in total work to fracture (6.3 ± 0.4 J vs. 0.7 ± 0.1 J vs. 1.7 ± 0.2 J, mean ± sem, wt vs. *oim* vs. *oim* + cells, P < 0.05) (Supplementary Figure 2D); increase in work from yield to fracture (7.3 ± 0.3 J vs. 0.5 ± 0.05 J vs. 1.5 ± 0.1 J, P < 0.01) (Supplementary Figure 2E); increase in % plastic work, as determined by the ratio of plastic/total load to fracture, (94.1 ± 0.7 vs. 30.6 ± 2.2 vs. 85.9 ± 1.9, P < 0.0001) (Fig. 3C); and maximum deflection (0.4 ± 0.01 vs. 0.2 ± 0.01 vs. 0.3 ± 0.01, P < 0.05) (Supplementary Figure 2F). Together these results indicate that AFSC transplantation decreased bone brittleness and increased the ability of the bones to deform before fracturing. Engraftment within the femoral epiphysis was positively correlated with total work to fracture (R^2^ = 0.68, P < 0.0001 deviation from zero) (Fig. 3D) and with bone stiffness (R^2^ = 0.8, P < 0.0001) (Fig. 3E), indicating that the low levels of donor cell engraftment may be a limiting factor to the benefits observed.

### Bones from transplanted mice were of better structural quality

**Figure 4:**
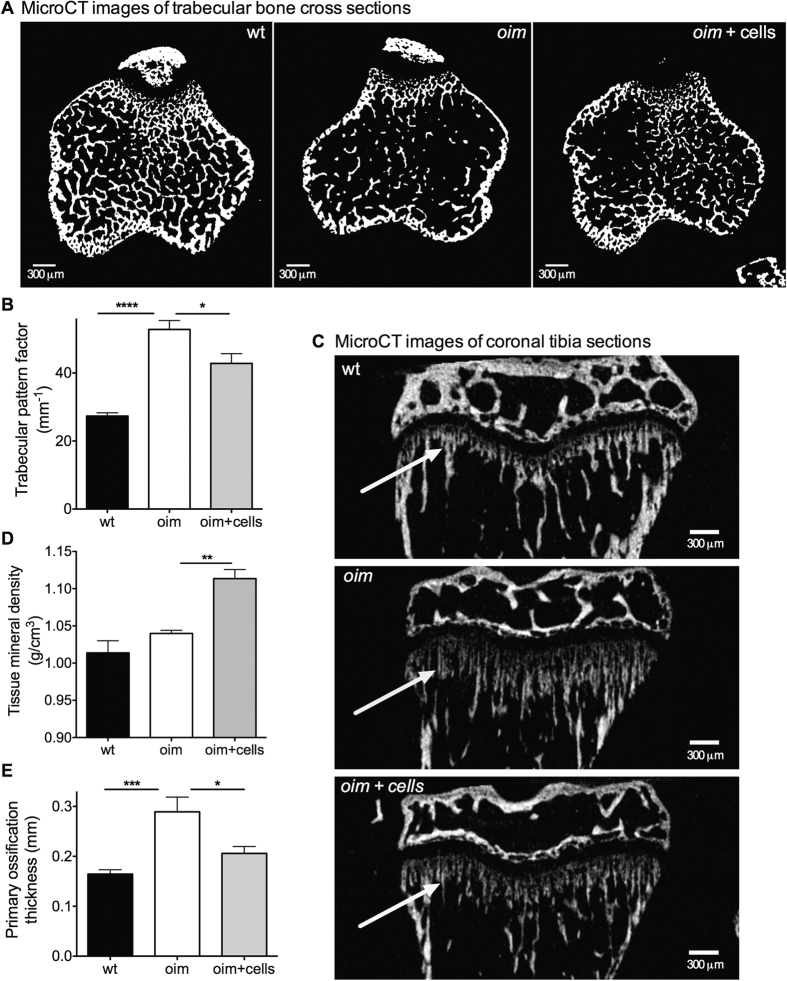
Effect of AFSC transplantation on bone microstructure. **(A)** Representative binarised microCT images of trabecular bone cross-sections at 0.25 mm below the tibial growth plate, obtained for 8-week-old wt, non-transplanted *oim* and transplanted *oim*. **(B)** Trabecular pattern factor. **(C)** Representative microCT image of coronal tibial sections of wt, *oim* and transplanted *oim*, with the primary ossification showed with an arrow. **(D)** Dot-plot of trabecular cortical tissue mineral density of 8-week-old wt (n = 6), *oim* (n = 6) and transplanted *oim* (n = 8) tibiae. All microCt parameters were analysed using analysis of variance (one-way ANOVA) followed by Bonferroni’s multiple comparison post hoc test. Data represent mean ± SEM. SEM: standard error of the mean.****P < 0.0001, ***P < 0.001 and * P < 0.05. wt: wild type non-transplanted mice, *oim*: *oim* non-transplanted mice, *oim+*cells: *oim* mice transplanted with AFSCs.

Increased resistance to mechanical load can be attributable to several factors, including the geometry of the bones and their composition. The microstructure of non-fractured tibia was analysed by microcomputed tomography (microCT), (representative scans showing increased trabecular connectivity following transplantation, Fig. 4A). Compared to wild type bones, *oim* bones showed reduced trabecular thickness (0.04 ± 0.001 mm vs. 0.03 ± 0.001 mm, wt vs. *oim*, mean ± sem, P < 0.001) (Supplementary Figure 3A); reduced trabecular number (3.2 ± 0.15 mm^−1^ vs. 1.3 ± 0.19 mm^−1^ vs., P < 0.0001) (Supplementary Figure 3B), and reduced Bv/Tv (12.2 ± 0.6% vs. 3.9 ± 0.7%, P < 0.0001) (Supplementary Figure 3C). AFSC transplantation did not modify trabecular thickness (0.03 ± 0.001) (Supplementary Figure 3A), but increased trabecular number (1.7 ± 0.2 mm^−1^) (Supplementary Figure 3B) and Bv/Tv (5.2 ± 0.7), albeit not reaching significance (P > 0.05) (Fig. 4B). Trabecular bone pattern factor, which provides a simple quantification of bone microarchitecture^20^, was higher in *oim* than in wild type mice (52.8 ± 2.6 mm^−1^ vs. 27.3 ± 0.9 mm^−1^, P < 0.0001), but was lowered by 19% in transplanted *oim* mice (42.8 ± 2.9 mm^−1^, P < 0.05), indicating that AFSCs transplantation improved architectural trabecular organization, i.e. orientation and connectivity of trabeculae (Fig. 4B).

### AFSCs increased mineral density of bone tissue

Figure 4C shows representative scans of coronal tibial sections. Bone mineral density (BMD), which relates to the amount of bone within a mixed bone-soft tissue region, was lower in *oim* than in wild type mice (0.03 ± 0.01 g/cm^3^ vs. 0.13 ± 0.01 g/cm^3^, *oim* vs. wt, mean ± sem, P < 0.0001), with a slight increase in transplanted mice not reaching significance (0.06 ± 0.01 g/cm^3^, P > 0.05) (Supplementary Figure 3D). In contrast to BMD, tissue mineral density (TMD), an index of the material density of the bone itself, does not differ between wild type and *oim* mice (1.01 ± 0.02 g/cm^3^ vs. 1.04 ± 0.004 g/cm^3^, P > 0.05), but was increased in transplanted *oim* (1.1 ± 0.01 g/cm^3^, P < 0.01) (Fig. 4D). Total porosity refers to volume fraction of the open and closed pores, and is directly related to bone density by (1-relative density)^27^,^28^. The percentage of total porosity was higher in *oim* bones than in wild type ones (7.2 ± 0.7% vs. 4.9 ± 0.4%, P < 0.05) but lowered in the bones of transplanted *oim* mice (5.4 ± 0.5%), albeit failing to reach statistical significance (Supplementary Figure 3E). The thickness of endochondral ossification in the primary spongiosa, which was higher in *oim* than in wild type bones (0.3 ± 0.03 mm vs. 0.16 ± 0.01 mm, P < 0.001), was also reduced in the bones of transplanted *oim* mice (0.2 ± 0.01 mm, P < 0.05), indicating increased skeletal maturity (Fig. 4B–D).

### AFSC transplantation increased mineral maturation

**Figure 5:**
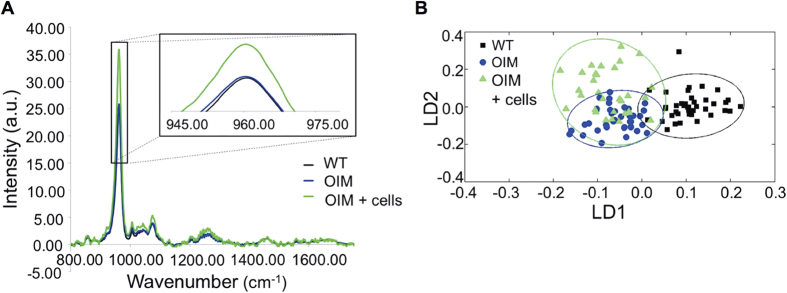
Mineral analysis of bones by Raman spectroscopy. **(A)** The average Raman spectrum for each group is shown, whilst the insert shows a close-up of the phosphate peak. **(B)** Raman spectra analysed with PCA-LDA: scores plot displaying results for wild type (black squares), non-transplanted *oim* (blue circles) and transplanted *oim* (green triangles) bones.

We next used Raman micro-spectroscopy to further characterise the overall biochemical fingerprint of each bone cohort. Results indicated a higher phosphate peak in the bones of transplanted *oim* mice compared to non-transplanted ones (Fig. 5A). Multivariate analysis confirmed a distinct separation between the wild type and *oim* groups (confidence interval of 95%), with transplanted *oim* bones overlapping with both group (Fig. 5B). Loading curves revealed that the differences along LD1 (separating wild type from *oim* groups) related to the phosphate peak, and changes between transplanted and non-transplanted *oim* bones (LD2) related to wavenumbers at 1256 (Amide III), 1002 (phenylalanine), 669 and 391 cm^−1^ (Supplementary Figure 4A). Analysis of crystal maturity, reflected by the width of the phosphate peak, revealed that transplantation was associated with higher crystal maturity compared to non-transplanted bones (96.3% vs. 95.3% compared to wild type bones referenced at 100%, P < 0.001) (Supplementary Figure 4B).

### AFSC transplantation up-regulated genes involved in osteogenesis and down-regulated those involved in inflammation, TGFβ and osteoclast differentiation

**Figure 6:**
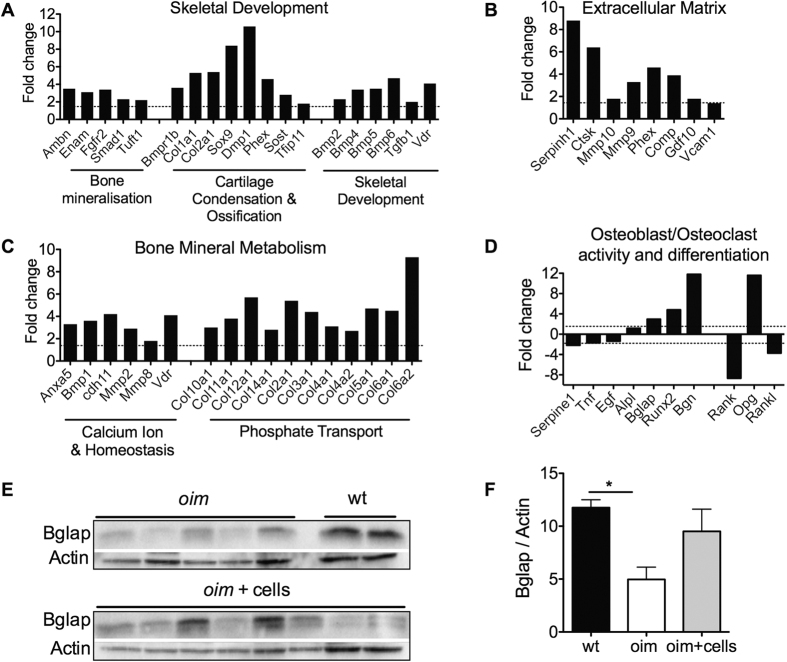
Gene expression in resident cells. Fold changes in the expression of mouse genes involved in **(A)** skeletal development, **(B)** ECM composition, **(C)** bone mineral metabolism, **(D)** osteoblast and osteoclast activity and differentiation, measured in the femoral epiphysis of 8-week old transplanted *oim*, compared to non-transplanted *oim* by gene array (n = 2). **(E)** Western blot of mouse osteocalcin (Bglap) in wt, *oim* and transplanted *oim* (cropped blot is shown, refer to Supplementary information for vision of full blot) and **(F)** quantification after normalisation with β-actin. Data were analysed using Student’s *t*-test. Data represent mean ± SEM. SEM: standard error of the mean. *P < 0.05. Wt: wild type non-transplanted mice, *oim*: *oim* non-transplanted mice, *oim* + cells: *oim* mice transplanted with AFSCs.

To understand the mechanisms by which AFSCs provided a countermeasure to bone fragility and improved bone quality, we analysed the phenotype of resident osteoblasts in the femoral epiphysis of transplanted and non-transplanted *oim* mice. Using quantitative RT-PCR, we showed an up-regulation (above 1.5-fold) for the genes coding for proteins facilitating long bone growth during skeletal development, in particular bone mineralization (i.e. Ambn, Enam, Fgfr2), ossification (i.e. Sox9, Dmp1, Phex, Sost), and skeletal development (i.e. Bmp2,4,5,6, Runx2 and Vdr), as well as a 5.5 fold upregulation of collagen type I, indicating that transplantation stimulated resident osteogenesis and collagen type I production (Fig. 6A). We also observed an up-regulation of expression for genes involved in bone extracellular matrix (ECM) formation, (i.e. Serpinh1, Ctsk, Mmp9 and Comp) (Fig. 6B), and those involved in homeostasis (i.e. Anxa5, Bmp1, Comp) and phosphate transport (Fig. 6C), indicating an increase in bone formation. Importantly, we also observed a downregulation of genes involved in stimulating osteoclastic bone resorption such as tumor necrosis factor (Tnf), which stimulates bone resorption by osteoclasts^29^,^30^; epidermal growth factor (EGF), which increases proliferation of osteoclasts precursors^31^, transforming growth factor-β 3 (TGFβ3), which is abundant in bone matrix and released as a consequence of osteoclast bone resorption; and Serpine1, a target of TGFβ^32^ differentiation; BGN (biglycan), which regulates osteoclasts activity through its effect on osteoblasts and their precursors by increasing their proliferation and maturation^33^. Genes expressed in osteoblasts, such as alkaline phosphatase (Alpl), osteocalcin (Bglap), Bmp2, and Runx2, were up-regulated in the bone of transplanted *oim* mice. The expression level of RANK Ligand (RANKL, Tnfsf11), which is produced by pre-osteoblasts and binds RANK receptors on the cell-surface of pre-osteoclasts to stimulate their differentiation, was decreased in transplanted bones, whilst the levels of osteoprotegerin (OPG, Tnfrsf11b), the decoy receptor for RANKL produced by mature osteoblasts, were increased, further indicating a stimulation of osteoblast maturation (Fig. 6D).

### AFSCs infusion increased protein levels of osteocalcin

To determine whether AFSCs transplantation stimulated the maturation of resident osteoblasts, we used western blot analysis to quantify the protein levels of osteocalcin (Bglap), which are exclusively expressed by mature osteoblasts. Results showed a significant reduction of osteocalcin expression in *oim* compared to wild type mice, confirming the impaired maturation of osteoblasts in OI^34^,^35^. We also found that the levels of osteocalcin expression were increased, although not reaching significance due to the high variability between animals (Fig. 6E,F).

## Discussion

The capacity of human fetal tissue-derived MSCs to reduce long bone fracture rate in mouse models of bone fragility has been reported previously, but transplantation failed to improve bone strength and only increased the plasticity of the bones^23^,^25^,^26^. Here we have established for the first time that human AFSCs can be used as countermeasure to bone fragility. Our data also revealed that the anabolic effects of the donor cells were mediated by the promotion of resident osteoblast maturation, leading to improved bone strength and bone quality.

**Figure 7:**
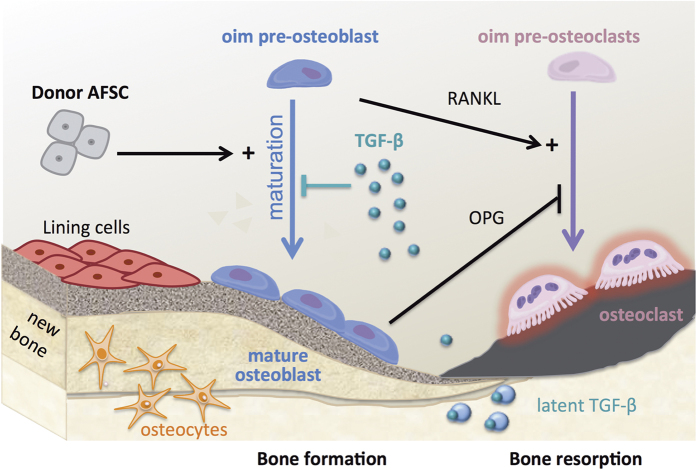
Model. From genetic defect to fractures in 5 steps: (1) *Oim* osteoblasts produce homotrimeric collagen type I fibres. (2) The presence of defective collagen in the extracellular matric ECM (orange) prevents the proper sequestration of TGF-β. (3) The excessive TGF-β signaling inhibits the differentiation of pre-osteoblasts into mature osteoblasts. (4) Thus, the majority of cells remain as pre-osteoblasts. (5) Since immature osteoblasts exhibit a stronger potential to support bone-resorbing osteoclast formation (pink) via production of RANKL, this leads to the stimulation of osteoclastogenesis. The transplantation of hAFSCs (6) improved bone strength and quality, by stimulating/promoting the maturation of *oim* pre-osteoblasts, which contributes to normalises bone remodelling.

Bone fragility in *oim* mice results from a decrease in quality and quantity of bone. Mutations in genes coding for Collagen type I chains or genes involved in their biosynthesis are responsible for the production of abnormal extracellular bone matrix (ECM), which prevents the proper sequestration of growth factors and cytokines in the ECM, such that it fails to convey the normal differentiation of pre-osteoblasts into mature osteoblasts^36^,^37^,^38^. As a result, only a small proportion of pre-osteoblasts attain the mature status. Since immature osteoblasts exhibit a stronger potential to support bone-resorbing osteoclast formation by the production of RANKL, impairment of osteoblast differentiation leads to increased bone resorption and further diminution of bone mass^36^,^37^. Therefore, promotion of resident osteoblast maturation, although not addressing the underlying genetic defect, shifts bone remodelling towards bone formation and normalises bone turnover (Fig. 7).

Transplanted AFSCs homed preferentially to bones where they remained for up to eight weeks after transplantation, localised at site of active bone formation under the primary spongiosa, expressed markers of mature osteoblastic commitment and produced Col1α2 protein, which is missing in the *oim* model^20^. These results show that AFSCs underwent osteoblast lineage differentiation *in vivo*, but the low level of donor cell engraftment indicates that donor-resident cell chimerism is unlikely to be directly responsible for the marked improvement of bone strength and quality, and may be more related to the capacity of AFSCs to migrate, engraft and differentiate when placed in an osteogenic environment. However, the presence of Col1α2 protein of donor origin in the bone extracellular matrix (ECM) may have a knock-on effect on bone quality. The low quantity and brittle quality of OI bones has recently been attributed to excessive TGFβ signaling, as a consequence of altered ECM^39^. The presence of abnormal collagen fibres is thought to prevent the proper sequestration of growth factors, cytokines and proteoglycans in the bone matrix, which fails to convey the normal signals that promote the recruitment of MSC to the bones and their differentiation into osteoblasts^36^,^37^. The presence of a minimal amount of normal collagen of donor cell origin in the bone matrix may be sufficient to increase the sequestration of the latent form of TGF-β and thereby decrease the levels of active TGF-β, such that the beneficial effect of donor cells is not mediated by direct osteoblast replacement but by their contribution to the ECM structure. In support of this hypothesis are the decreased levels of TGF-β target Serpine1 observed in the bones of transplanted mice.

An alternative explanation for our findings is that the anabolic effect of transplanted AFSCs was not mediated by their osteoblastic differentiation but the cells directly influence resident osteoblast maturation by releasing factors that stimulate their maturation. We found that genes involved in inflammation, such as TNFα and EGF, were down-regulated in transplanted mice, suggesting the possibility that the stimulation of endogenous osteoblast differentiation may be mediated by anti-inflammatory effects. In both situations, increasing the number of mature osteoblasts contributes to normalise bone turnover by lowering the differentiation and activity of osteoclasts. As a result, the collagen formed is still largely composed of abnormal homotrimeric fibers, but is produced in greater amounts. In line with this, we found an upregulation of collagen type 1 in the bones of transplanted animals.

Although it is necessary to further elucidate the trophic factors responsible for the beneficial effects of AFSCs, this study suggests that modulating osteogenesis can be an effective therapeutic target to counteract bone fragility and normalise bone remodelling.

## Methods

### Ethics statement

The healthy donors who provided the amniotic fluid in this study provided written informed consent in accordance with the Declaration of Helsinsky. The ethical approval given by the Research Ethics Committees of Hammersmith & Queen Charlotte’s Hospitals (08/H0714/87) in compliance with UK national guidelines (Review of the Guidance on the Research Use of Fetuses and Fetal Material (1989), also known as the Polkinghorne Guidelines. London: Her Majesty’s Stationery Office, 1989: Cm762) for the collection of fetal tissue for research. All experimental protocols were approved by the UK Home Office guidelines (PPL 70/6857), and The Institutional Licensing Committee of Imperial College London and University College London. All methods were carried out in accordance with relevant guidelines and regulations.

### Cell culture

Human amniotic fluid mesenchymal stem cells (AFSCs) (passage 5–8), were isolated from the amniotic fluid at 12 weeks of gestation (normal pregnancy), which was obtained under ultrasound guidance. The cells from three individuals with normal karyotype were selected for c-KIT expression and expanded (10^4^ cells/cm^2^) on plastic culture dishes without feeders in Dulbecco’s modified Eagle’s medium (DMEM-HG) (Invitrogen) supplemented with 10% fetal bovine serum (Biosera), 2 mM L-glutamine, 50 IU/ml penicillin and 50 mg/ml streptomycin (Gibco-BRL), at 37 °C in a 5% CO_2_ incubator.

### *In vitro* differentiation

Cells were differentiated along the osteoblast lineage for 4 weeks in DMEM-LG supplemented with 10 mM β-glycerophosphate, 0.2 mM ascorbic acid and 10^−8^ M dexamethasone, then fixed in 10% formalin. Cells were differentiated along the adipocyte lineage over 2 weeks in DMEM supplemented with 0.5 mM hydrocortisone, 0.5 mM isobutyl methylxanthine and 60 mM indomethacin, then fixed and stained with oil red O. Cells were differentiated along the chondrocyte lineage over 2 weeks in DMEM-LG supplemented with 0.01 μg/ml TGF-β3, 0.1 μM dexamethasone, 0.17 mM ascorbic acid, 1 mM sodium pyruvate, 0.35mM L-proline, 1% ITSS, 50 μg/ml Linoleic Acid (reagents from Sigma), then cells were fixed in and stained with alcian blue (2%).

### Flow cytometry

AFSCs were detached, washed with FACS buffer (PBS supplemented with 1% BSA, Sigma), and stained with primary antibodies CD73-PE, CD90-APC and CD105-FITC (Miltenyi) for 1 hour at 4 °C. Cells were then washed again with the FACS buffer and analysed by FACScalibur flow cytometry (Becton Dickinson).

### Animals and stem cell transplantation

Heterozygous male and female (B6C3Fe a/a-Col1a2^oim^/Col1a2^oim^) mice (Jackson Laboratory) were housed in individual ventilated cages at 21 °C with a 12:12-hour light dark cycle. Offspring were genotyped by sequencing the *oim* fragment then homozygous and wild type colonies established. Progeny were weaned at 30 ± 1 day and culled at 8 weeks of age. AFSCs (10^6^ cells resuspended in 20 μl of cold PBS) were injected intra-peritoneally (i.p.) into 3–4 day-old *oim* neonates and mice were culled for analysis when they were 8 week old. We noted no variability between different isolated AFSC specimens in terms of phenotype (data not shown) and donor cells injected in *oim* mice were from a single donor. Controls comprised age-matched non-transplanted o*im* and wild type mice.

### Counting of fractures

Fractures in femurs, tibias, and humeri were assessed at 8 weeks of age by determination of callus formation (n = 168 transplanted and n = 156 *oim* control). Three independent observers blinded to transplantation status assessed the number of mice with at least one long bone fracture, as well as the fracture incidence (number of fractured bones/total bones assessed). Fracture incidence was assessed by Chi-squared with Yates correction and to one degree of freedom. Differences with a P-value of <0.05 were considered significant.

### Mechanical testing

Three-point bending tests were performed on 8 week-old non-fractured femurs, fresh-frozen and thawed prior to testing. Bones were placed on two supports 10 mm apart and tested to failure using a standard material testing machine (5866 Instron, Norwood, MA, USA). Femurs were loaded at the mid-diaphysis in the anterior-posterior direction with a deflection rate of 50 μm/s. Force-deflection curves were analysed with a custom program (Matlab, MathWorks Inc, MA, USA) to measure the bending stiffness (S: slope of the linear elastic deformation, N/mm), the yield load (Fyield: limit between the elastic and plastic deformation, N) and ultimate load (Fult: maximum force sustained, N). The plastic (post-yield) behaviour was assessed by the ratio of plastic on total work to fracture (Rp/tW, i.e. ratio of the area under the curve from the yield point to the fracture point over the total area under the curve), total work to fracture (maximum force sustained prior to fracture, mJ), work from yield to fracture (mJ), and maximum deflection (deflection at fracture in mm). P values were calculated using analysis of variance (one-way ANOVA) followed by Bonferroni’s multiple comparison post hoc test. Differences with a P-value of <0.05 were considered significant. Data were expressed as mean ± SEM (standard error of the mean).

### Microcomputed tomographic analysis

Tibiae were isolated from 8-week old transplanted (n = 9), non-transplanted *oim* mice (n = 6) and wt mice (n = 6), and fixed in 10% neutral buffered formalin for 24 h. Samples were then washed in phosphate buffered saline and stored in 70% ethanol until scanning. All scans were performed using a Skyscan 1172 μCT scanner (Bruker, Kontich, Belgium). The bones were scanned at 50 kV and 200 μA using a 0.5 mm aluminium filter and a resolution of 4.3 μm. To analyse the trabecular bone, a region of interest of the length of 1 mm was selected 0.25 mm below the growth plate of the tibia. To analyse cortical porosity and tissue mineral density, a 0.5 mm long region of interest was selected 2.25 mm below the growth plate. The images were reconstructed using the Skyscan NRecon software and the following cortical and trabecular morphometric parameters were calculated using the Skyscan CT Analyzer (CTAn) software: percent bone volume (BV/TV) (%), trabecular thickness (Tb.Th) (mm), trabecular number (Tb.N) (mm^−1^), trabecular pattern factor (Tb.Pf) (mm^−1^) and total cortical porosity (Ct.Po) (%). Bone mineral density and tissue mineral density were measured using 0.25 and 0.75 g/cm^3^ calcium hydroxyapatite calibration phantoms (Bruker) as a reference. P values were calculated using analysis of variance (one-way ANOVA) followed by Bonferroni’s multiple comparison post hoc test. Differences with a P-value of <0.05 were considered significant. Data were expressed as mean ± SEM (standard error of the mean).

### Raman spectroscopy

Humeri (n = 10 per group) were dissected, wrapped in PBS soaked gauze and kept at −80 °C until use. Raman spectra were acquired using a Renishaw inVia micro-spectrometer (Renishaw plc, Gloucestershire, UK), equipped with an 830 nm laser. 5 spectra of 600 s (10 s × 60 accumulations) were acquired step-wise from along the anterior aspect of each bone. The spectra were baseline corrected using a polynomial to remove fluorescence and normalised to the Amide I peak (1660 cm^−1^). The phosphate to collagen ratio was calculated by dividing the height of the phosphate peak (960 cm^−1^) by the height of the Amide I peak (1660 cm^−1^); similarly, a second ratio was calculated using the proline (920 cm^−1^) and hydroxyproline (850 cm^−1^) peaks. Mineral crystal maturity was estimated by calculating the inverse of the full width of the phosphate peak at half the height (1/FWHH)^40^. Principal component analysis-linear discriminant analysis (PCA-LDA) was applied to the data to look at the spread of the intra- and inter-category variance and identify the wavenumbers, and subsequent biochemicals, associated with any segregation in the data. The output is displayed as two plots, one scores (scatter) and one loadings (pseudo-spectrum). The former shows each spectrum as one dot, or score, and provides a visualisation of the spread of the data, whereby proximity of the scores is directly related to biochemical similarity. The loadings curves are the axes from the scores plot and allow identification of the wavenumber, and therefore biochemical, responsible for any segregation along a particular axis.

### Gene expression analysis

Total RNA was extracted from 8-week-old mice femoral epiphysis, using TRIzol (Invitrogen), followed by RNA clean-up (RNeasy Qiagen) and cDNA synthesis using RT^2^ First Strand Kit (Qiagen). Gene expression was examined by RT^2^ Profiler mouse PCR arrays (Qiagen) and analysed according to the manufacturer’s instructions (n = 2 mice per group).

### Engraftment

Donor cell engraftment was measured by quantitative real-time PCR as previously described^23^. Femurs of the same mice were dissected and separated into epiphysis (n = 20) and diaphysis (n = 20). The bone marrow was flushed from the diaphysis with PBS and analysed (n = 20). Donor cell engraftment was also measured in brain, thymus, spleen, liver, lungs and kidney (n = 10 for each group). RNA was then extracted using TRIzol (Invitrogen) followed by cDNA synthesis with M-MLV reverse transcriptase (Promega). To calculate donor cell engraftment quantitative real time PCR (qRT-PCR) was performed using SYBR green dye (Applied Biosystems) and the ABI Prism 7700 Sequence Detection System with human specific (human) and human-mouse non-specific (human:mouse) β-actin primers (human:mouse F: 5′-GCT CCT CCT GAG CGC AAG TA-3′ R: 5′-GAT GGA GGG GCC GGA CT-3′; human F: 5′-CTG GAA CGG TGA AGG TGA CA-3′, R: 5′-AAG GGA CTT CCT GTA ACA AT GCA-3′). Human:mouse chimerism was estimated as the ratio of human β-actin to total human and mouse β-actin in the total cDNA sample to give the 2^−DCt^ value. Samples were considered positive with a human specific β-actin Ct above 36 at a threshold of 0.13ΔRn. Negative controls were non-transplanted *oim*. P values were calculated using analysis of variance (one-way ANOVA) followed by Bonferroni’s multiple comparison post hoc test. Differences with a P-value of <0.05 were considered significant. Data were expressed as mean ± SEM (standard error of the mean).

### Immunohistochemistry

8-week-old mice tibias were dissected, decalcified in 10% EDTA pH 7.4 and subsequently embedded in paraffin. 4 μm sections were cut, de-paraffinized in xylene, and rehydrated. Heat induced antigen retrieval was performed on a hot plate with citrate buffer at pH 6 followed by serum-free blocking agent. The presence of differentiated donor cells in transplanted mice was determined using human-specific osteopontin (Vector) primary antibody overnight followed by secondary antibody tagged to FITC.

### Western blot

Proteins were extracted from mouse femurs adding RIPA buffer (1% Nonidet P-40, 0.5% sodium deoxycholate, 0.1% sodium dodecyl sulfate (SDS), and 0.004% sodium azide) to ground bone. Proteins were run on an 8% SDS-PAGE, transferred to nitrocellulose, blocked with milk, and stained with osteocalcin (15 kDa) primary antibody (Clontech), HRP-linked anti-rat IgG secondary antibody (Abcam) and collagen 1 alpha 2 (130 kDa) primary antibody (Santa Cruz Biotechnology), HRP-linked anti goat IgG secondary antibody (Santa Cruz Biotechnology) and followed by enhanced chemiluminescence detection (Thermo Scientific). The loading control used was β-actin (43 kDa) (Abcam). *P*-values were calculated using Student’s *t*-test. Differences with a P-value of <0.05 were considered significant. Data were expressed as mean±SEM (standard error of the mean).

## Additional Information

**How to cite this article**: Ranzoni, A. M. *et al*. Counteracting bone fragility with human amniotic mesenchymal stem cells. *Sci. Rep.* **6**, 39656; doi: 10.1038/srep39656 (2016).

**Publisher's note:** Springer Nature remains neutral with regard to jurisdictional claims in published maps and institutional affiliations.

## Supplementary Material

Supplementary Information
