# Supplementary material for: Counteracting bone fragility with human amniotic mesenchymal stem cells
Source: Sci Rep. 2016 Dec 20;6:39656. doi: 10.1038/srep39656 (PMC5171815; doi:10.1038/srep39656)
Supplement: Supplementary Information [file srep39656-s1.pdf]

Counteracting Bone Fragility with Human Amniotic Mesenchymal Stem Cells

Anna M. Ranzoni[1], Michelangelo Corcelli[1], Kwan-Leong Hau[1], Jemma G. Kerns[2], Maximilien Vanleene[3], Sandra Shefelbine[4], Gemma N. Jones[5], Dafni Moschidou[1], Benan Dala-Ali[6], Allen E. Goodship[6], Paolo De Coppi[7], Timothy R. Arnett[8], Pascale V. Guillot[1]*

[1] *Institute for Women's Health, University College London, London, UK*

[2] *Lancaster Medical School, Lancaster University, Lancaster, UK*

[3] *ONCOLille, Regional University Hospital of Lille, Lille, France*

[4] *Department of Mechanical and Industrial Engineering, Northeastern University, Boston MA, USA*

[5] *Institute of Reproduction and Developmental Biology, Imperial College London, London, UK*

[6] *Institute of Orthopaedics and Musculoskeletal Science, Royal National Orthopaedic Hospital, University College London, Stanmore, UK*

[7] *UCL Great Ormond Street Institute of Child Health, University College London, London, UK*

[8] *Department of Cell & Developmental Biology, University College London, London, UK*

* Corresponding author: Pascale V. Guillot, Institute for Women's Health, Maternal and Fetal Medicine Department, University College London, 86-96 Chenies Mews, London, WC1E 6HX, United Kingdom.

E-mail p.guillot@ucl.ac.uk

Supplementary Information

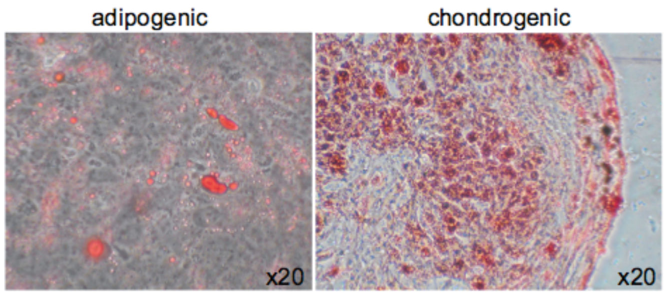

Supplementary Figure 1 | Differentiation of AFSC. *In vitro* differentiation of AFSCs down the chondrogenic, adipogenic and osteogenic pathways.

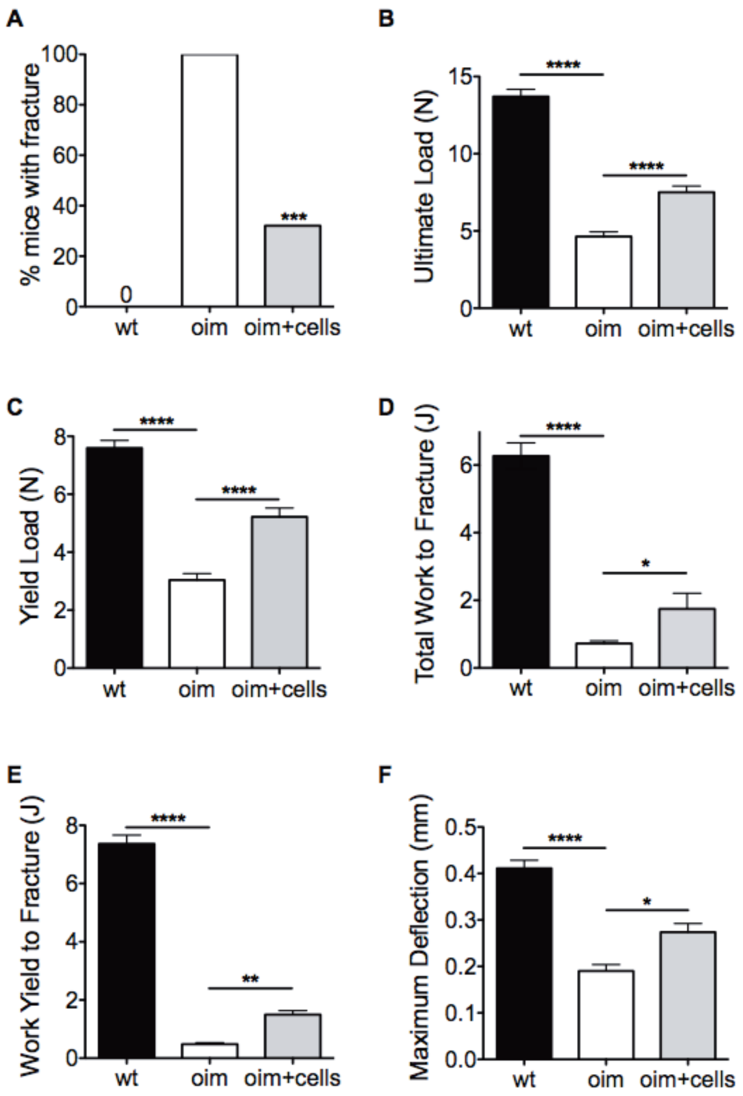

Supplementary Figure 2 | Effect of AFSC transplantation on bone mechanical properties. (A) Percentage of 8-week-old mice with long bone fractures (n=30 wt, n=26 *oim* and n=28 *oim* + cells) assessed by Chi-squared

with Yates correction and to one degree of freedom. Differences with a P-value of <0.05 were considered significant. **(B)** Dot-plot of three-point bending load-deflection curves until fracture obtained for 8-week-old wt (n=20), *oim* (n=17) and *oim* transplanted (n=13) femurs: ultimate load and **(C)** yield load. **(D)** Dot-plot of three-point bending load-deflection curves for total work to fracture, **(E)** work from yield to fracture, and **(F)** maximum deflection. Three-point bending data were analysed using analysis of variance (one-way ANOVA) followed by Bonferroni's multiple comparison post hoc test. Data represent mean ± SEM. SEM: standard error of the mean. **** P<0.0001, *** P<0.001, ** P<0.01 and * P<0.05. Wt: wild type non-transplanted mice, *oim*: *oim* non-transplanted mice, *oim*+cells: *oim* mice transplanted with AFSCs.

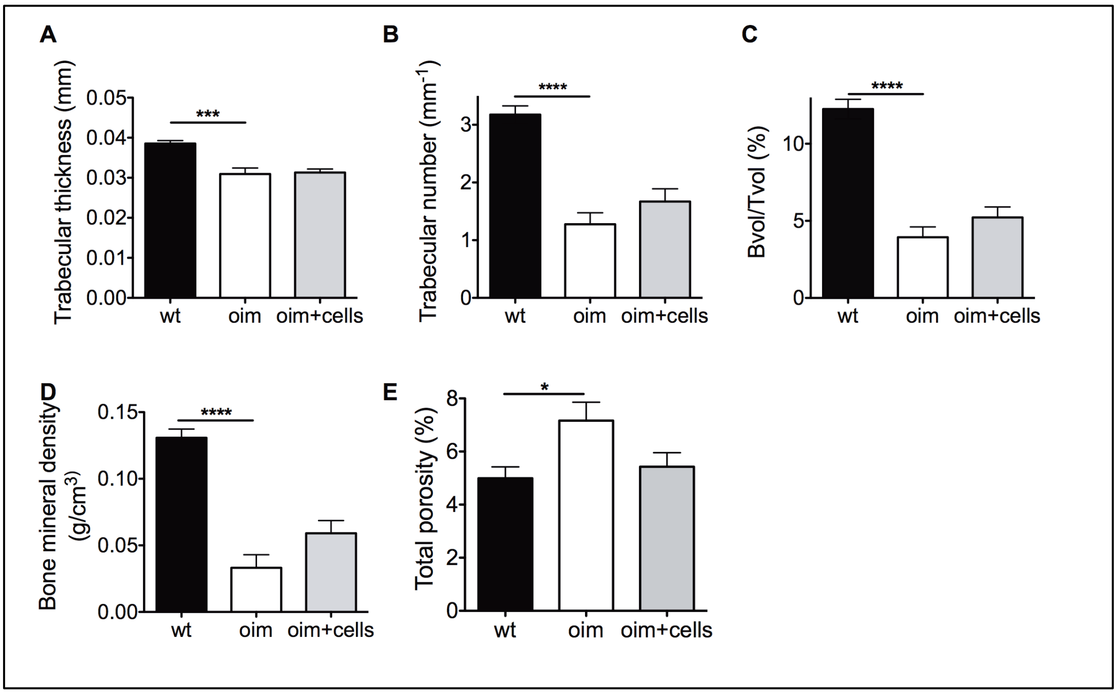

Supplementary Figure 3 | Effect of AFSC transplantation on bone microstructure. (A) Dot-plot of microCT trabecular morphological parameters, in wt (n=6), non-transplanted *oim* (n=6) and transplanted *oim* (n=9) mice: trabecular thickness, **(B)** trabecular number, and **(C)** bone volume/tissue volume. **(D)** Dot-plot of trabecular bone mineral density and **(E)** total cortical porosity of 8-week-old wt (n=6), *oim* (n=6) and transplanted *oim* (n=8) tibiae. All microCt parameters were analysed using analysis of variance (one-way ANOVA) followed by Bonferroni's multiple comparison post hoc test. Data represent mean ± SEM. SEM: standard error of the mean.**** P<0.0001, *** P<0.001 and * P<0.05.

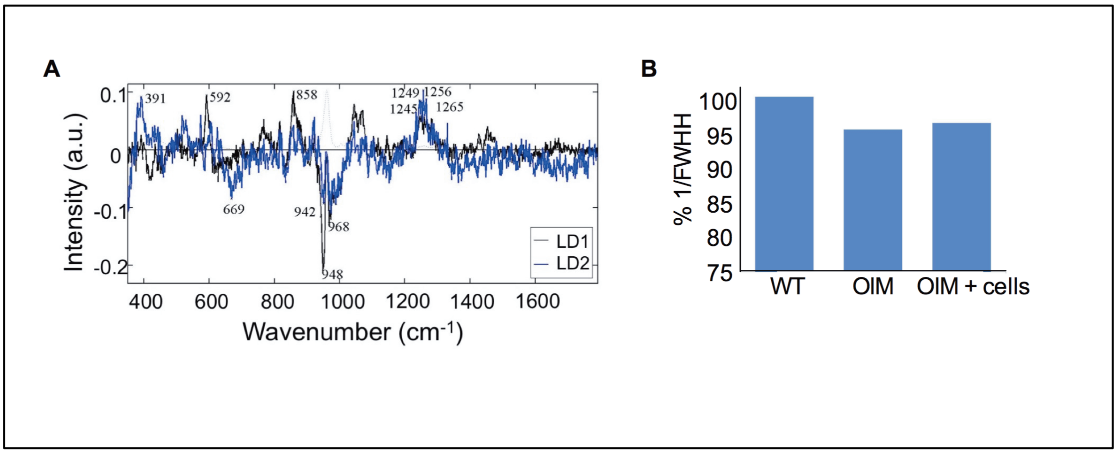

Supplementary Figure 4 | Mineral analysis of bones by Raman spectroscopy. (A) Loadings plot corresponding to Figure 5A, with the wavenumbers contributing most significantly labelled; the dashed line in the background is the average spectrum from the whole spectral dataset. **(B)** Average crystallinity of each cohort of bones, using wild type as a reference at 100%.
